# Governance and Power Across Intersecting Value Chains: The Case of South African Apples

**DOI:** 10.1007/s10551-023-05337-9

**Published:** 2023-02-15

**Authors:** Margareet Visser, Matthew Alford

**Affiliations:** 1 Labour, Development and Governance Research Unit, Faculty of Law, Kramer Building, Middle Campus, University of Cape Town, Cape Town, South Africa; 2 Alliance Manchester Business School, University of Manchester, Room 5.059, Booth Street West, Manchester, M15 6PB UK

**Keywords:** Global value chains, Governance, Power, Ethical trade

## Abstract

A prevailing focus of global value chain (GVC) analysis has been on the dominance of highly consolidated Northern retailers over suppliers in the global South. The rise of regional and domestic value chains (RVCs/DVCs) within the Global South which intersect with GVCs, has been found to involve private governance by Southern lead firms. However, we have limited insight into the implications of this changing value chain context for the role of public governance, or different groups of workers. South African fruit provides a rich example of rapid shifts in RVCs/DVCs governed by different private and public actors. The following two questions are addressed: How is the public–private governance of labour standards evolving in the context of RVCs and DVCs that intersect with GVCs? What are the implications for workers operating across different value chains? Conceptually, the paper draws on GVC analysis of governance and power, to examine the governance of labour standards across intersecting value chains. Our analysis highlights the intentional and unintentional mechanisms through which power and standard-setting are diffused away from Northern lead firms to a wider array of public and private actors operating across RVCs/DVCs. While existing analysis of governance and power focuses on singular GVCs, our study highlights diffusion of power across intersecting value chains, with significant and uneven implications for the public–private governance of labour standards. Our findings carry significant ethical implications for the governance of labour standards, as end-markets continue to shift South.

## Introduction

Global value chain (GVC) analysis has shed important light on how transnational retailers in the global North ‘govern’ GVCs by coordinating sourcing, setting the commercial conditions and standards for suppliers based in the global South (Gereffi, 1994; Gereffi et al., 2005). This dynamic, referred to as ‘private governance’, has been subject to considerable academic attention along two distinct yet interrelated themes: first, commercial inter-firm relations between buyers and suppliers (Gereffi, 1994; Gereffi et al., 2005); and second, lead-firm enforcement of product quality, production process and social standards as a key dimension of these sourcing arrangements, enabling them to retain control over production (Nadvi, 2008). More recent literature highlights the central role of public actors in governing GVCs (Alford & Phillips, 2018; Mayer & Phillips, 2017). This strand of research has shed useful light on how private standards and public regulations interact, with (positive and negative) implications for workers in GVCs (Alford, 2020; Bartley, 2018; Locke, 2013). However, research into public–private governance to date has mainly focused on GVCs governed by Northern lead firms (Pasquali & Alford, 2021; Pasquali et al., 2021b).

Recent research shows that the volume of South-South trade is now surpassing North–South trade (Horner & Nadvi, 2018), coupled with an expansion of lead firms from the global South operating within and across their own regions (Barrientos et al., 2016; Neilson et al., 2014; Staritz et al., 2011). These shifts increasingly provide suppliers in developing countries the opportunity to serve various chains oriented to different end-markets across the global North and South—a dynamic referred to as ‘polycentric trade’ (Horner & Nadvi, 2018). This changing geography of value chains holds crucial economic and social implications for suppliers and workers, including how and by whom they are governed (Kaplinsky et al., 2011; Tessman 2018). It also raises profound questions for existing global value chain (Gereffi et al., 2005; Ponte & Sturgeon, 2014) and wider business ethics literatures (Dentoni et al., 2018; Detomasi, 2007; Reinecke & Ansari, 2016; Schrage & Gilbert 2021), which have focused primarily on the ethical behaviours and governance strategies of Northern lead firms. The increasing relevance of Southern end-markets creates a more complex and uneven ethical terrain that warrants much more sustained academic attention. This paper therefore responds to Horner and Nadvi’s (2018, p. 231) call for further research into governance dynamics in a context of polycentric trade:

…we are still in the nascent stages of understanding how lead firms from the Global South organize their value chains, how much they outsource and what factors shape their chain linkages. Moreover, while standards are clearly present in Southern markets, we know less about what drives them and how they relate to (and differ from) the pre-existing Northern ones.

For example, previous research indicates that sourcing in regional and domestic value chains (RVCs/DVCs) may emphasise product standards over labour and environmental concerns due to their significance in driving competitiveness (Barrientos et al., 2016; Horner & Nadvi, 2018; Knorringa & Nadvi, 2016; Pickles et al., 2016).^1^ But the evidence is mixed and we lack understanding of the implications of rising polycentric trade for public governance, including its interaction with private governance and implications for workers. This paper therefore addresses the following questions: *How is the public–private governance of labour standards evolving in the context of RVCs and DVCs that intersect with GVCs? What are the implications for workers operating across different value chains?*

Analytically, we advance a governance-power framework for analysing private–public governance in a context where GVCs, RVCs and DVCs overlap. We draw on Gereffi et al.’s (2005) seminal typology of private governance to help understand the different *market, modular, relational, captive* and *hierarchical* linkages that exist between lead firms and their suppliers, and the extent to which lead firms control and coordinate production through the enforcement of standards (including labour) across different end-markets. Drawing on existing literature on the role of public governance (albeit in a context of North–South GVCs), helps us account for the central regulatory role of the state and public–private governance interactions across different value chains (Alford, 2016; Alford & Phillips, 2018). Such an approach contributes to existing analysis of public governance in GVCs led by Northern lead firms (Alford, 2016; Alford & Phillips, 2018) by accounting for expanding RVCs and DVCs in the global South. We further posit that *power* is a key dimension of governance in a context where diverse value chains intersect (Dallas et al., 2019; Pasquali et al., 2021a) involving a range of lead firms, suppliers, state and civil society actors. As we will observe, the significance of RVCs and DVCs alongside GVCs leads to a diffusion of power away from Northern lead firms’, restricting their ability to coordinate suppliers and directly enforce private standards. Power therefore becomes increasingly diffuse and collective, shared by a broader range of private and public actors beyond Northern lead firms (Pasquali & Alford, 2021; Pasquali et al., 2021a). Crucially, our analysis will reveal that the particular nature and form of power diffusion (and public–private governance mode that follows) varies across RVCs and DVCs, with uneven implications for workers.

Empirically, we examine horticulture value chains wherein global and domestic retailers play an increasingly important role within the global South (Das Nair, 2018; Henson & Reardon, 2005). Private governance in this context involves enforcement of standards covering product quality and food safety, best practice agricultural production processes and labour conditions. Our particular focus is on South Africa’s apple sector, itself a core component of the country’s wider agricultural industry. Apples, traditionally exported to Northern retailers in the UK and Europe via GVCs, are now increasingly sold regionally and domestically. South Africa is arguably at the heart of Sub-Saharan Africa’s (SSA) retail revolution, with domestic (South African) supermarkets gaining more market share. Domestic supermarkets source apples through DVCs that intersect with both Northern GVCs and informal markets (similar trends have been observed in Kenya’s horticultural sector—see Krishnan, 2018; Pasquali et al., 2021a). Producers have also made important inroads into SSA via RVCs, with the bulk of trade occurring through a mix of (primarily) arm’s length market arrangements alongside direct sales to retailers.

On the one hand, these dynamics have driven ‘convergence’ of private labour standards (including those of the Ethical Trading Initiative (ETI) base code and GlobalGAP) as standards from Northern GVCs spill over into certain RVCs and DVCs (Pasquali et al., 2021a; Pickles et al., 2016). On the other hand, the rise of RVCs and DVCs has diffused power away from Northern lead firms, and widened the multiplicity of private and public actors involved in (or absent from) the governance of labour. As we will argue, these contradictory dynamics have generated uneven regulatory protection for South African fruit workers.

The research was carried out in 2018–21. In the first phase, we mapped the commercial and governance dynamics of GVCs, RVCs and DVCs, drawing on secondary data and key informant interviews with private (20), state and parastatal (5), civil society (6) actors and one academic with extensive research experience in this context. The total number of interviews was 32. Following this, we purposively selected fresh fruit producers to gain an in-depth understanding of labour standard requirements across intersecting value chains. Ten sites were examined in total. One of these had a high exposure to GVCs serving Northern lead firms, RVCs and DVCs (P1); five had a low exposure to Northern lead firms sourcing via GVCs, but a high exposure to South African lead firms sourcing via DVCs (P2, P6, P7, P8, P9); three had no exposure to Northern lead firms sourcing via GVCs, despite exporting 50% of their crop (P3, P4, P5), and of their domestic sales, two of these three sold at least 20% to South African retailers via DVCs (P3, P4); one did not export any product via GVCs or RVCs, selling the bulk of their product domestically via arm’s length market arrangements (P10) (see Table 4). This second research phase comprised in-depth interviews with producers (10) and semi-structured interviews with workers (60), both permanent (34) and temporary (26), equating to a total of 70 interviews during this period. The entire project involved 102 interviews in total.

Specific themes included in interviews varied by respondent, depending on their role and perspective in relation to our overarching research questions. Such variations depended on whether respondents were positioned to formulate and implement (i.e. government agencies and lead firms); monitor (civil society organisations (CSOs); or were on the receiving end of the public–private governance initiatives under study (producers and farm workers). The core aim was to better understand how public–private governance of labour standards played out across value chains, and the implications for workers. Workers were canvassed on key aspects of labour and health and safety legislation/standards that impacted their everyday working lives. During the analysis phase, interviews with different value chain actors were coded, extracted and entered into a consolidated document which was then condensed and refined. Key variables per producer operation were also entered into a table to obtain a ‘bird’s eye overview’ of producers’ market integration and labour management strategies. Worker interviews (and the farms they worked on) were captured in an excel-spreadsheet, allowing us to filter and cross-correlate against key variables. Through iterative analysis, key themes and trends were pin-pointed.

The paper proceeds as follows. The next section draws upon GVC conceptions of governance and power to frame our analysis of the public–private governance of labour standards in a context of polycentric trade. The third section investigates these issues through the case study of South African fruit, wherein we examine governance and power dynamics across the GVCs, RVCs and DVCs under investigation. The fourth section explores in greater depth how private–public governance plays out across intersecting value chains, and the implications for workers. The fifth and final section reflects analytically on the findings and concludes.

## Private–Public Governance of Labour Standards: from GVCs to ‘Polycentric Trade’

### Private–Public Governance in GVCs

In line with the acceleration of economic globalisation since the early 1990s, a number of frameworks have sought to conceptualise how transnational trading networks are structured and governed (Coe & Yeung, 2015; Coe et al., 2008; Gereffi, 1994; Gereffi et al., 2005; Henderson et al., 2002). The global value chain (GVC) framework has provided crucial insights into ‘private governance’ and how lead firms coordinate and control sourcing arrangements with their geographically dispersed supply base. Different conceptualisations of private governance have evolved over time. One early typology distinguished between buyer and producer-driven chains to explain differential power relations between lead firms and suppliers across different sectors (Gereffi, 1994). Horticultural value chains are generally referred to as ‘buyer-driven’, governed by large, consolidated retailers based in developed country locations (Dolan & Humphrey, 2004). As a condition of supply, producers in developing countries must comply with lead firms’ governance demands, including adherence to stringent private standards relating to product quality, the production process, social and environmental conditions (Nadvi, 2008; Pasquali et al., 2021a).

Subsequent governance frameworks sought to account for the increasing complexity of products and knowledge exchanged through GVCs. Gereffi et al.’s (2005) seminal typology identifies five distinct governance types, depending on differing levels of codifiability and constraints on supplier capabilities. At one extreme, *hierarchical* governance structures arise when product specifications are difficult to codify and supplier capabilities constrained and risks of non-compliance high. At the other extreme, arm’s length *market* exchanges arise when specifications are simple, easy to codify and costs associated with direct coordination are unwarranted. In-between these structures of full integration and arm’s length transactions are *modular* chains, where suppliers produce goods in adherence with buyers’ specifications and standards leading to low switching costs for both parties; *relational* chains, in which product specifications cannot be codified, transactions are complex, and supplier capabilities are high, resulting in high switching costs for both parties; and *captive* chains, where suppliers’ capabilities are low and they need to be closely monitored by a small subset of buyers, leading to high switching costs for suppliers.

Gereffi et al.’s (2005) fivefold governance typology has facilitated important analysis of lead firms’ ability to demand supplier adherence with product, process and social standards (Nadvi, 2008). However, this typology has received criticism for its narrow conceptualisation of the regulatory framework (which excludes public regulation) in which firms operate (Bair, 2008). In reality, the power dynamics underpinning interactions between lead firms and suppliers are heavily shaped by the institutional and social contexts in which global production takes place. Lead firms are not the only actors influencing the governance of GVCs (Coe et al., 2008). Numerous studies have revealed that non-firm actors—and particularly nation states—play a central regulatory role in determining who gains and loses from participation in global production (Alford, 2016; Alford & Phillips, 2018; Mayer & Phillips, 2017).

This has given way to considerable interest in ‘private–public’ governance and how private standards and state regulations intersect to shape social (and environmental) conditions in GVCs (Alford, 2020; Bair, 2017; Bartley, 2018; Locke, 2013). Findings from this literature show that private standards can ‘complement’ national labour regulation, by enforcing compliance with labour legislation and thus providing more protection to workers (Amengual & Chirot, 2016). Similarly, Gereffi and Lee (2016) call for combinations of private (CSR regimes and corporate codes of conduct), public (national governmental laws and regulations) and civil society (trade union and NGO activity) initiatives through what they term ‘synergistic governance’, to secure improved social conditions in GVCs.

The above insights into private–public governance highlight that power in GVCs is a multidimensional concept spanning lead firms’ ‘coordination and governance’ activities and the ‘regulatory’ activities of states and civil society (Dallas et al., 2019; Gereffi & Lee, 2016; Ponte et al., 2019). Following Dallas et al.’s (2019) recent conceptualisation, power in GVCs can be *dyadic*, as is the case in relations between lead firms and their suppliers; and/or *collective*, referring to government regulation and civil society influence on commercial chain actors. Power can also be *direct*, ‘when an actor (or collective) wielding power and those who are objects of it are relatively easy to identify’ and ‘the exertion of direct power is ….intentional and the goals of powerful actors are usually more transparent’ (Dallas et al., 2019, p. 674). In contrast, power is *diffuse* when those exerting the power are less transparent, their goals not necessarily intentional and change occurs due to demonstration and rising trends leading to normalisation and acceptance of standards (as in the case of quality conventions or best practices) (Dallas et al., 2019, p. 673). As depicted in Table 1, this two-by-two typology generates four types of power exercised in GVCs: bargaining power (direct/dyadic), demonstrative power (diffuse/dyadic), institutional power (direct/collective) and constitutive power (diffuse/collective) (Dallas et al., 2019; Grabs & Ponte, 2019). As noted by Grabs and Ponte (2019), far from being mutually exclusive, these types of power can overlap, combine and influence each other, as will be demonstrated in the case of South African apples.

**Table 1:** A typology of power in GVCs

	Direct transmission	Diffuse transmission
Dyadic actor constellation	*Bargaining power* (operates in firm-to-firm relations with various degrees of asymmetry)	*Demonstrative power* (operates through informal transmission mechanisms along value chains); e.g. private standards
Collective actor constellation	*Institutional power* (operates through government regulation, multi-stakeholder initiatives or other institutionalised forms)	*Constitutive power* (operates through broadly accepted or taken-for-granted norms, conventions and best practices

*Source* Adapted from Grabs and Ponte (2019, p. 810)

In line with this conceptualisation, lead firms’ bargaining position is greater in more captive and relational chains, comprising close inter-firm relations and monitoring underpinned by direct and dyadic power (Pasquali et al., 2021a). Conversely, power dynamics become more diffuse in arm’s length market-based and modular linkages, and/or through ‘decentralised collaboration among loosely or unaffiliated actors’ (Dallas et al., 2019, p. 673). However, while existing analyses of power helpfully elucidate the diffusion of power along singular value chains (see Dallas et al., 2019; Grabs & Ponte, 2019), little attention has been given to the possible diffusion of power *across* intersecting value chains, and the implications of this for public–private governance. Moreover, it is important to note that literature on public–private governance and power is predominantly focused on GVCs coordinated by Northern lead firms, which is problematic given the changing geography of end-markets associated with polycentric trade.

### Private–Public Governance in an Era of Polycentric Trade

The rise of polycentric trade suggests that ‘differences in governance may well emerge’ within particular sectors supplying different end-markets (Horner & Nadvi, 2018, p. 224). This requires analysis of governance to ‘include overlapping procurement strategies and standards requirements by global and regional buyers that exists across (rather than just within) the chains in which they intersect’ (ibid: 224). Yet, research is still at a nascent stage in understanding how private standards and public regulations co-exist and interact across GVCs, RVCs and DVCs (Horner, 2016; Horner & Nadvi, 2018; Pasquali et al., 2021a; Pickles et al., 2016). This is due to the fact that analysis of governance in a context of polycentric trade has focused on private as opposed to public governance.

While the vast majority of existing GVC analysis in the horticulture sector is predicated on flows of products and knowledge between Northern buyers and suppliers in the Global South, increasing evidence shows that producers supply lead firms and buyers across different regional and domestic markets (Barrientos et al., 2016; Das Nair, 2018). Previous research highlights that lead firms in RVCs and DVCs are often located at the intersection between coordinated GVCs and less formal markets; effectively sourcing from GVC-oriented suppliers and those selling into local markets (Pasquali et al., 2021a). Some studies show that certain ‘multi-chain’ suppliers negotiate this complex scenario by serving multiple buyers across GVCs, RVCs and DVCs, making strategic choices around the volume and quality of products sold and which standards to adhere to, thus enhancing their bargaining power with different buyers (Navas-Aleman 2011; Ouma, 2010; Pasquali et al., 2021b).

All of this has implications for private governance: at this stage lead firms sourcing via coordinated GVCs are still better able to govern their dedicated supply base relative to lead firms in RVCs and DVCs who source through heterogeneous channels (Navas-Aleman 2011; Pasquali & Alford, 2021). Previous studies demonstrate that sourcing through open markets via arm’s length transactions is subject to limited private governance, insofar as there is no direct power of lead firms over suppliers. This paper draws on Dallas et al.’s (2019) conceptualisation of governance and power, to examine how and by whom labour standards are governed in a context of intersecting value chains. In doing so, it advances recent investigation of governance and power in a context of polycentric trade (Pasquali et al., 2021a) by extending analysis to consider the implications of these changes for workers.

Interactions between lead firms and their suppliers in GVCs are more likely to be characterised by dyadic and direct power, given that lead firms occupy a relatively strong bargaining position and are able to govern suppliers directly through private standards (Dallas et al., 2019; Pasquali et al., 2021a). Concomitant with the rise of polycentric trade, where RVCs and DVCs increasingly intersect with GVCs and arm’s length markets, the power of lead firms in GVCs becomes more diffuse as suppliers have access to a wider range of buyers. Moreover, the power of domestic and regional lead firms is comparatively less dyadic and direct (Pasquali et al., 2021a), as they use more heterogeneous sourcing channels. This includes modular, direct linkages with dedicated suppliers alongside market-based, arm’s length transactions absent of direct coordination (Hanlin & Kaplinsky, 2016; Kaplinsky, 2000). Overall, in a situation characterised by a wider range of buyers operating across heterogeneous sourcing channels (including arm’s length), regional and domestic lead firms’ dyadic power over suppliers becomes constrained and their capability to directly coordinate suppliers is reduced (Pasquali & Alford, 2021). This leads to what Pasquali et al., (2021a:9) term a ‘private governance void’, which in certain contexts (such as Kenyan horticulture) can precipitate a shift towards more active public governance in regulating RVCs and DVCs.

This paper builds on these insights by extending analysis of public–private governance and power in a context of polycentric trade, to consider the implications of changing power relations for different groups of workers operating across different value chains. Previous research highlights that in GVCs, lead-firm demands for high-quality, low-cost goods with faster turnaround have exacerbated the use of temporary workers in GVCs (Kidder & Raworth, 2004; Standing, 2009). In contrast to permanent workers employed on formal contracts, temporary workers are generally employed on less secure, short-term contracts; experience poorer wages and working conditions (Barrientos & Smith, 2007); and are less protected by private codes of conduct (Barrientos, 2008; Hale & Wills, 2007), national labour legislation (Alford, 2016); and civil society representation (Barrientos, 2013). But we lack understanding of how differential private–public governance of intersecting value chains affects the working conditions of different groups of workers.

The above discussion clearly reaffirms the need to avoid simple generalisations about the governance of GVCs, RVCs and DVCs, including who stands to win or lose from participation in them. It is clear that each case—including our examination of South African apples—requires a nuanced understanding of the diverse governance structures and changing power relations between private *and* public actors, which change over time as value chains intersect. Our core motivation is to consider the implications of this for the governance of labour standards and different groups of workers incorporated into intersecting GVCs, RVCs and DVCs. We now turn to explore these issues empirically through analysis of the South African apple sector.

## Private–Public Governance Across Intersecting Apple Value Chains in South Africa

### Private Governance

#### Global Value Chains: Modular and Consolidated

Until the late 1990s, the bulk of South African apple exports were destined for Northern GVCs, and specifically EU and UK markets. This trade pattern was shaped by South Africa’s legacy of colonialism and geographical factors. Being based in the Southern hemisphere allowed South African apple producers to supply Northern markets during the latter’s off-season when supplies were low, providing them with an important resource rent. Yet, Meyer & Breitenbach (2004, p. 26) argued that this trade pattern ‘underline[d] the vulnerability of South African apple exports to changes in the EU’s demand for apples’. Essentially, South African suppliers stood in a captive relationship with Northern buyers. Until 1997 this vulnerability was shielded by a powerful state-controlled deciduous fruit marketing board (Unifruco, later Capespan), which negotiated en-bloc on behalf of all apple export farmers.

**Fig. 1:**
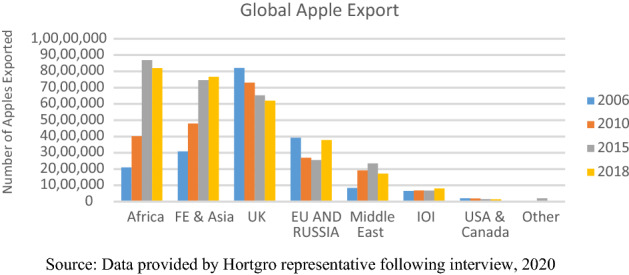
Changes in South African apple export markets over time: 2006 to 2018. Source Data provided by Hortgro representative following interview, 2020

State protection of South African apple producers ended abruptly with deregulation of the agricultural sector in 1997. Marketing boards were closed, fragmenting the collective power of producers who now had to negotiate individually with a few, large consolidated Northern supermarkets. South African producers still found themselves in a captive relationship with their Northern buyers, but without the protection of marketing boards: up to 1999 72% of South Africa’s total apple export crop was primarily sold to lead firms in the EU and UK (See Fig. 1) (Meyer & Breitenbach 2004, p. 29). Northern supermarkets leveraged their oligopolistic position to exert direct power over suppliers with whom they had a dyadic relationship, by setting the terms of trade and exerting private standards on producers, driving up production costs (Barrientos & Visser, 2012). Such commercial pressures felt by producers were compounded following the state’s extension of labour regulation to the agricultural sector and introduction of a minimum wage in 2003, which increased farm labour costs.

In 2003 all South African apple exporters were also required to be certified against GlobalGAP, the private standard promoting good agricultural practices, food safety, environmental standards, but also occupational health and safety standards (Greenberg, 2003). This was followed by private standards for packhouses, relating to quality assurance and food safety (e.g. Dutch HACCP; Food Safety System Certification (FSSC) required by German retailers; and BRC required by British retailers). In 2008 UK supermarkets, soon to be followed by EU supermarkets, began enforcing the UK’s Ethical Trading Initiative’s (ETI) code on their South African suppliers. The ETI code is based on core International Labour Organisation (ILO) principles, designed to protect workers and ensure decent work in GVCs.

The post-deregulation period led to several bankruptcies followed by a process of farm consolidation in the fruit sector (Du Toit, 2004). Concurrently, EU and UK retailers were also consolidating, by eliminating category managers in favour of centralised trading platforms and directly trading with fewer, bigger suppliers who could consistently supply large volumes of quality fruit (Barrientos & Visser, 2012), reduce retailers’ transaction costs and decrease reputational risks involving food safety and human rights scandals. Over time, trade relationships between Northern lead firms and fewer, more consolidated apple producers changed from captive to modular, but with UK and EU retailers still exercising direct and dyadic power over their suppliers.

Despite changing relationships between producers and Northern lead firms sourcing via GVCs, the latter continue to exercise considerable pressure on producers’ profit margins. For instance, Visser and Ferrer (2015) found that from 2003 to 2013 producers never received more than 27.5 and 29.4%, respectively, of the final retail price for apples and pears. UK and EU retailers’ increasing commercial pressures on suppliers and their simultaneous enforcement of private standards have created a ‘pincer effect on producers, who have responded by restructuring the labour force’ (Barrientos & Kritzinger 2003, p. 92).

This has led to the retrenchment of permanent workers and their replacement with temporary workers employed on insecure, short-term contracts (Barrientos & Visser, 2012). Over the past decade, this process of casualisation has intensified, particularly in the labour-intensive apple and pear subsectors, where temporary workers comprise 70—80% of workers during peak season (Visser & Ferrer, 2015). This trend has severely impacted job security, with temporary, fixed-term apple workers less likely to be unionised, for fear their contracts will not be renewed. A combination of union-underrepresentation in the agricultural sector; weak enforcement of labour rights by the Department of Employment and Labour (DoEL); and civil society pressure on Northern lead firms, has driven the latter to enforce private social standards. Yet, partly due to being in a slightly more secure trading relationship with their Northern buyers, South African apple suppliers recently began to contest UK and EU retailers’ demand for private social standards. Apple producers successfully advocated for self-regulation, leading to the establishment of the Sustainability Initiative of South Africa (SIZA), giving suppliers more control over the code-making and certification process (Alford et al., 2021). At the same time, SIZA plays an important function in protecting South African fruit farms’ access to international markets, by diminishing risks of further exposés about poor working conditions (Alford et al., 2021; Visser & Ferrer, 2015).^2^

To become less beholden to Northern lead firms, *all* South African apple producers, including smaller, less well-resourced apple farmers, also began developing new markets. This drive became critical in 2010, with the development of Dynamically Controlled Atmosphere (DCA) technology, enabling long-term storage of apples without serious quality losses. DCA technology enables Northern hemisphere apple producers to supply UK and EU retailers with apples throughout the year, while eroding the resource rent of Southern suppliers. It prompted the latter to rapidly turn to Africa, a growing market on their doorstep. By 2015 Africa surpassed the UK in terms of volumes of South African apples received (see Fig. 1) and by 2020 it constituted the major market for South African apple farmers, taking 27% of all exported apples. Of all apple exports to Africa, 70% is shipped to West Africa and 30% to East Africa (Interview, South African retailer, 2019). Market diversification, including into Africa, has contributed to less captive and more modular relationships between South African apple producers and Northern lead firms, as switching costs to new regional and domestic buyers are reducing. On face value, this potentially erodes the dyadic and direct power of Northern lead firms over their South African apple suppliers.

The UK and Europe, however, remain important markets taking, respectively, 18% and 9% of South African apple exports (See Fig. 1). For South African apple suppliers, these markets have changed from commodity to high value markets, procuring the most sought-after varieties, including Pink Lady® and other patented bi-colour varieties (See Table 2 below). Stringent private product and food safety standards demanded by Northern lead firms constitute high entry barriers for most apple suppliers, leading to rents for large, well-resourced, consolidated suppliers who can scale these barriers.

**Table 2:** Pink Lady® versus Cripps Pink apple prices in different markets (2019)

Destination market	Cripps Pink (non-branded variety of Pink lady®)	Quality	Average price per kilogram
Jhb FPM	Cripps Pink	Local class 1	R3.99 to R7.57
Local supermarkets	Cripps Pink/Pink Lady®	Local class 1/EU Class 2	R8 to R9
German discount supermarkets	Pink lady®	EU class 2	R17
UK supermarket	Pink lady®	UK supermarket standards (varies per supermarket)	R25

*Source* Author interviews with apple producers

#### Regional Value Chains: Market-Based and Modular

For suppliers who cannot meet the high private standards of Northern lead firms, RVCs offer a welcome reprieve. A major benefit is that African buyers purchase commonly grown apple cultivars such as Golden Delicious, that have become unpopular in Europe and the UK (Hortgro, Tree Census 2018). Hence, approximately 70% of South Africa’s Golden Delicious crop is exported to West Africa (Interview, fruit exporter, 2019). One producer-exporter commented that South Africa’s glut of Golden Delicious and West Africa’s preference ‘fitted [South African producers] like a glove’ (Interview, fruit producer, 2020). Likewise, in East Africa, South African producers sell less-popular Granny Smith apples as well as Cripps Pink, the generic equivalent of Pink Lady® apples that fails to meet branded colour specifications. While stressing that African buyers are quality conscious, producer-exporters remarked that in more economically suppressed regions, buyers are prepared to buy high grade, but smaller sized or less coloured apples that would otherwise be sold for processing at much lower prices (Interviews, three producer-exporters, 2019–20).

Despite Africa’s growing population and middle class (Signe, 2018) and predictions that the continent will become the new retail frontier (McKinsey, 2015; Weatherspoon & Reardon, 2003) with supermarkets driving the expansion of African horticultural value chains (e.g. Barrientos et al., 2016; Pickles et al., 2016; Weatherspoon & Reardon, 2003), the majority of fresh apples are still sold on wet markets. In some countries more than 50% of *retail*—let alone apple sales—remains informal, with levels in Benin, Tanzania and Nigeria exceeding 65% (Medina et al., 2017). One South African retailer estimated it to be as high as 80% in some African countries (Interview, South African retailer, 2019). Commented another South African retailer:

When a vessel arrives with apples from South Africa in Nigeria, there is normally anything from 30 to 50 containers of apples on it: maybe about 10% of that apples would be ours; the rest would be other importers that are more informal, who mostly supply the informal markets’ (Interview, South African retailer, 2019).

Because of this, one South African supermarket does not sell fresh food in its regional stores (Interview, South African retailer, 2019); and a second only sells fruit through its African franchisees, who are directly responsible for sourcing apples from South Africa. Only two South African retailers sold apples regionally through their consolidated supply chains (Interviews, two South African retailers, 2019–20). They load shipping containers either at their distribution centres (DCs) or at apple producers’ packhouses/DCs and export it to their DCs in Africa. Retailers frequently complained about logistical bottlenecks, bribery and long delays at border posts and harbours (Interviews, four South African retailers, 2019–20). Trade data for exports into Kenya—which, like South Africa, has experienced significant supermarket penetration—suggests that currently, major South African producer-exporters, rather than supermarkets, are driving *horticultural* trade into Africa (See Table 3).

**Table 3:** South African actors exporting apples into Africa

South African actors exporting apples to Africa	Value (USD) of exports/imports
Producer-exporter 1	1 597 656
Producer-exporter 2	1 335 912
Producer-exporter 3	1 326 615
Producer-exporter 4	940 850
Specialised African exporter 1	936 693
Specialised African exporter 2	242 938
Supermarket 1	32 701
Supermarket 2	24 767

*Source* Authors’ compilation based on Kenyan Revenue Authority (2018) dataset

This situation has arisen only in the last ten years. Before that, specialised South African exporters were the main intermediaries between South African apple producers and African importers, who in turn supplied smaller retailers, the hospitality industry and consolidators and informal traders in wet markets. However, since DCA technology has pushed major producer-exporters out of UK and EU markets, they have begun trading directly with African buyers, thus bypassing South African exporters specialising in trade with Africa (Interviews, three producer-exporters, 2019–2020). Two producer-exporters sold 20% of their total crop in Africa in 2019 and one of them sold 80% directly to African buyers. One of these producer-exporters established a presence in Kenya to better ply this market. Most of this trade could be described as market-based, arm's length trade, with a myriad of buyers having little direct power over major, consolidated producers. While most buyers in Africa demand fairly high product standards relating to quality assurance, they do not require any agricultural process or social standards (Interview, fruit exporter, 2019).

As a general rule of thumb, South African producers integrated into GVCs try to export the maximum volume of fruit into EU and UK markets, due to the high prices offered (see Table 2). Producers supplying Northern lead firms therefore treat their whole crop as if destined for GVCs, and must ensure their entire crop is certified against stringent private product, agricultural process and social standards. When the same producers sell their apples into RVCs, Northern lead firms’ private standards spill over into African markets, regardless of their level of formality. But not all South African producers selling fruit into Africa are integrated into GVCs. The same South African specialised exporters who have been bypassed by their former clients are now sourcing from smaller packhouses and producers who are not necessarily integrated into GVCs. It is not a given that these apples are certified against any private social standards. Hence, when African buyers sourcing apples via RVCs use the same suppliers as Northern lead firms, they benefit from the latter’s private governance arrangements through a process of standards diffusion. However, where they buy from suppliers not integrated in Northern GVCs, no private standards may apply.

#### Domestic Value Chains: Market-Based and Modular

The third major outlet for South African apples are domestic value chains (DVCs), into which approximately 25% of the national apple crop is sold (Hortgro, 2019).^3^ DVCs comprise two main types of trade relationships between producers and their buyers: modular trade, wherein producers sell their apples via coordinated DVCs to South African retailers (domestic lead firms) exercising dyadic, direct power; and arm’s length trade, of which trade on the National Fresh Produce Markets (NFPMs) is the most important. Buyers on NFPMs include wholesalers, independent retailers, hawkers and informal cross-border traders, but also supermarkets. Transactions on the NFPMs are indirect and no private standards apply. However, the fact that approximately 30% of apples sold on NFPMs are supplied by major producer-exporters also serving GVCs, means Northern lead firms’ private standards also spill over into NFPMs.^4^ Similar to RVCs, private standards are thus diffused by multi-chain producers integrated across intersecting value chains.

The most important domestic market of South Africa’s five major multi-chain apple producers is South African retailers, whose share of fresh fruit sales has grown from 10% in 2002 to 50% in 2016 (Barrientos et al., 2016).^5^ To illustrate this point, two South African retailers have set up their own DCs in major centres to directly source large and consistent volumes of apples from multi-chain producers (Interviews, two South African retailers, 2019). Representatives of South African retailers stressed that they deliberately sourced most of their apples from large, multi-chain producers to reduce their reputational risks: they knew these producers were certified against Northern lead firms’ private product, food safety and social standards. The process by which South African retailers intentionally ‘piggy-back’ on the stringent social standards of their Northern counterparts, reflects a form of demonstrative power, wherein Northern lead firms’ private standards become diffused into DVCs over time.

Unlike Northern supermarkets who sell to a relatively homogenous consumer community, South African retailers cater to a range of customers—from the highest to lowest income groups—through different store formats ranging from flagships to discount stores. When retailers cater for lower income customers, they not only compete with other supermarkets, but also with smaller retailers and hawkers, who source fruit predominantly from NFPMs. Not to be outcompeted on price, domestic retailers also buy from the NFPMs. While they stressed they only do so when experiencing unforeseen supply shortages, some also bought apples on the NFPM during the peak season when apples prices were low (Interviews, two South African retailers; one NFPM employee, 2019–20). Franchisees of supermarket groups also regularly source from NFPMs, as franchisors do not prescribe from which suppliers franchisees should source. Franchisors are acutely aware of the risks this presents. To protect their brand, one retailer requires as a minimum that franchisees put in place documented control measures, to record all sourcing of fresh food and enhance traceability.

At times, domestic lead firms also trade directly with producers *not* integrated into GVCs. This happens, for instance, when South African retailers want to purchase the first fresh apples of the season, but also when sourcing for their discount stores. One retailer commented that its discount chain sourced fruit through a different corporate entity than its flagship stores (Interview, South African retailer, 2019). In these instances, retailers apply their own private product quality and agricultural process standards (which are lower than those of Northern lead firms). Yet, even these standards would sometimes be relaxed depending on the need for special product, or to allow new suppliers to ease into compliance with standards (Interview, South African retailer, 2019). Domestic lead firms therefore unevenly apply private product quality and agricultural process standards. *None* (bar one exception) consistently enforce private social standards on their suppliers.^6^

The stringent demands associated with supplying apples via GVCs, together with increased opportunities in RVCs and DVCs, lead to the differential integration of suppliers across chains depending on their relative capabilities and resources. Well-resourced, major multi-chain suppliers serve GVCs, RVCs and DVCs. Smaller, less well-resourced suppliers are excluded from GVCs due to the high entry barriers posed by Northern lead firms’ private standards, and are more likely to be inserted into RVCs and/or DVCs (Interview, fruit industry representative, 2020).

That many South African apple producers may be switching away from GVCs is suggested by the fact that 45% of deciduous fruit producers (of which apple producers are the major subset) are *not certified* by SIZA, the dominant private social certification scheme of South Africa’s fresh fruit sector.^7^ This 45% could potentially be supplying RVCs and/or DVCs, where no social standards are consistently enforced (with the exception of one South African supermarket sourcing domestically). The rise of RVCs and DVCs increases producers’ bargaining power, by making them less beholden to their buyers and reducing switching costs. While private social standards at this stage are diffused into RVCs and DVCs by those producers simultaneously integrated into GVCs, the switch to RVCs and DVCs could also lead to a move away from private social standards. However, public governance provides a crucial (if not variegated) layer of regulatory protection that is differentially applied across intersecting value chains, which we will now turn to discuss.

### Public governance

The South African state’s post-apartheid policy included implementation of a raft of comprehensive labour regulation designed to protect farmworkers, deemed particularly vulnerable to exploitation under apartheid (Du Toit, 2004). Consequently, farmworkers are protected by a suite of labour legislation. This includes the Labour Relations Act (1995) (LRA), which provides the legal framework to enable unionised workers to negotiate with their employers for improved working conditions that surpass legal minimum requirements. However, due to extremely low levels of unionisation in the agricultural sector, the LRA has limited influence on farmworkers’ employment conditions or bargaining power. As a result, the Minister of Employment and Labour drafted Sectoral Determination 13, which sets minimum working conditions for all agricultural workers on a sector-wide basis. Farm workers are also protected by the Occupational Health and Safety Act (OHSA, 1993).

While labour regulation provides farmworkers with an important layer of protection on paper, the state’s institutional power to enforce it is extremely weak. The DoEL’s Inspection and Enforcement Service (IES) is the government agency charged with enforcing labour legislation, but suffers from severe resource constraints (Alford, 2016; Visser & Ferrer, 2015). To illustrate this point, the ratio of labour inspectors to workers in the Western Cape was 1:16090 in 2013/14, markedly out of step with the ILO’s advised ratio of 1:10000 (DoEL 2017, p. 49). Consequently, the labour inspectorate is largely unable to extend coverage to geographically dispersed agricultural workplaces found in remote areas, including fruit farms located in the Western Cape.^8^ IES is, however, aware of the impact of private audits of social standards by Northern lead firms’ and SIZA (which demand compliance with national labour legislation) in driving the ‘complementary’ enforcement of state labour regulation (Interview, IES official, 2021).

Complementarity between private standards and public law is also observable in the enforcement of health and safety standards on exported fruit. Given that the major focus of certain private standards (such as the packhouse standards BRC, HACCP, and the farm-level standard GlobalGAP) is on food safety and traceability, the South Africa’s Perishable Product Export Control Board (PPECB), responsible for enforcing minimum public product and food safety standards pertaining to the export of all fresh fruit the country, accepts these private standards in lieu of its own food safety regulation (GN707).^9^ Of these standards, GlobalGAP is the least demanding and has become the quasi-minimum criterion for South African fruit exports. By accepting GlobalGAP—which contains extensive occupational health and safety standards—PPECB simultaneously reinforces a private standard demanded by Northern lead firms, as well as South Africa’s Occupational Health and Safety Act (OHSA).

## Public–Private Governance Across Intersecting Value Chains: Implications for Workers

Examination of public–private governance across intersecting value chains reflects a complex scenario, underpinned by multiple and interlayered forms of power. In relation to private governance, Northern lead firms enforce stringent private standards directly via dyadic and modular linkages with a dedicated supply base. In contrast, both RVCs and DVCs reflected more heterogeneous sourcing mechanisms, involving combinations of modular and direct linkages between lead firms and their suppliers, alongside arm’s length sourcing arrangements observed in NFPMs. Significantly, lead firms sourcing via regional and domestic VCs (with one exception) only require compliance with product quality and agricultural food safety standards (such as HACCP, BRC and GlobalGAP) but not social standards (such as SIZA). However, diffusion of private social standards from GVCs to RVCs and DVCs occurs when ethically certified ‘multi-chain’ sites supplying GVCs sell their products into RVCs/DVCs, resulting in private social standards inadvertently spilling over into RVCs/DVCs. It also happens when South African retailers sourcing via DVCs intentionally source apples directly from ethically certified suppliers integrated into GVCs, thus ‘piggy-backing’ on Northern lead firms’ social standards in order to mitigate risks. This scenario reflects the diffuse transmission of *demonstrative* power, wherein social standards are informally transmitted not along, but *across* GVCs to RVCs and DVCs.

In relation to public governance, and in line with previous studies, we found that while the national labour legislative framework is relatively strong, the institutional power of the DoEL to enforce it is extremely weak. Private social standards demanded by Northern lead firms therefore effectively ‘complement’ the enforcement of, and compliance with, public regulations on certain sites linked to GVCs (Bartley, 2018; Locke, 2013). Complementarity goes one step further in relation to PPECB’s proactive enforcement and acceptance of GlobalGAP (a food safety standard reinforcing South Africa’s OHSA), in lieu of the state’s public food safety standards regulating fresh fruit exports. In this case, occupational health and food safety standards/regulations benefiting farms workers are diffused into RVCs through a process of *constitutive* power.

Table 4 provides a summary of farm size, market orientation, standards compliance and worker composition. We refer to this information throughout the following discussion of our farm-level fieldwork findings, to illustrate how differential integration into GVCs, RVCs and DVCs shapes the private–public governance of labour standards.

**Table 4:** Producers’ chain integration and certification against key private standards

Producer	Hectare apples	Packhouse on farm	Nr of workers (% of permanent workers)	Exports as % of total sales	Domestic sales as % of total sales	% of exports to UK & EU retailers via GVCs	% of exports to Africa via RVCs**	% of domestic sales to South African retailers directly via DVCs	GlobalGAP	SIZA
1	1060	Y	3420 (46%)	77%	23%	36%	25%	98%	Y	Y
2	72	Y	570 (28%)	50%	50%	50%	25%	75%	Y	Y
3	104	Y	255 (100%)	60%	40%	Seldom	5%	20–25%	Y	N
4*	0	Y	203 (73%)	50%	50%	Seldom	20%	20%	NA	N
5	64	Y	256 (49%)	60%	40%	Seldom	5%	0%	Y	N
6*	0	Y	190 (5%)	37%	63%	8%	8%	95%	NA	Y
7	33	N	100 (10%)	37%	63%	8%	8%	95%	Y	Y
8	14	N	27 (26%)	37%	63%	8%	Unknown	95%	Y	Y
9	12	N	30 (27%)	37%	63%	8%	8%	95%	Y	Y
10	10	N	44 (91%)	0%	100%	NA	NA	Seldom	Y	N

*Packhouse only, and therefore GlobalGAP not required

**Note that the majority of sales via RVCs are directly to a variety of wholesalers and market traders, but not to supermarkets. The balance of exports—not directed to GPN or RPN—is mainly exported to the Middle and Far East

Our fieldwork suggests that ethical certification alone was not a reliable indicator of compliance with private and public labour standards. More reliable was the *combination* of *ethical certification* and *consistent supply of a significant share of their crop to Northern lead firms*, who exert dyadic and direct power over producers, and whose loss as a client would present a *major economic risk* for producers (such as P1) tightly integrated into GVCs. As such, length of certification was a key indicator of more compliant working conditions. However, as elaborated below, we also found that levels of private–public standards compliance varied both by *type of working condition* (i.e. measurable standards vs enabling rights) and *group of worker* in question (i.e. permanent vs temporary).

Only one producer in our study exported a major component of its crop to Northern lead firms via GVCs. P1, a major, multi-chain producer-exporter that supplied more than a third of its crop via GVCs, also did its own marketing and sold directly to Northern lead firms. While P2 and P6-9 were also integrated into GVCs, they sold a much smaller proportion of their total crop via this channel to Northern lead firms (see Table 4). Compared to other producers, P1 had by far the most to lose economically from non-compliance and could not afford to risk its brand reputation. P1 had therefore made a concerted effort to comply with Northern lead firms’ private social standards, evidenced by the appointment of an internal compliance manager ten years prior. The compliance manager conducts regular internal audits on P1’s farms, and provides certification and human resources support both on P1’s own farms and independent producers exporting under its brand. Commented P1’s compliance manager:

Initially, the [private social] audits stood in front of us like a mountain. It was a struggle to get buy-in from the suppliers. At the time they did not pay workers’ sick leave or night allowance; housing was not up to standard and up until five years ago some still did not pay the minimum wage. (Interview, compliance manager, P1, 2021).

To protect its brand, P1 gave non-compliant producers exporting three months to correct their non-compliances following audits or face being dropped. However, monitoring and enforcing compliance on all its supplier sites, especially those that were geographically remote, remained a challenge.

Where producers simultaneously supplied GVCs, RVCs and DVCs, private social standards *supposedly* spill over into RVCs and DVCs because they in any event have to comply with social standards demanded by Northern lead firms sourcing via GVCs. According to major stakeholders interviewed, producers aim to export most of their crop, and at least some part thereof via GVCs due to the premium prices received (See Table 2). This is reflected by the fact that most producers in our sample (with the exception of P10) had *some* exposure to GVCs. Yet, with the exception of P1 and P2, most of these producers exported less than 10% of their product via GVCs (P6, 7, 8, 9), or to such an insignificant degree they were unaware what percentage of their exported harvest was being sold into GVCs (P3, P4, P5).

Smaller producers attributed their limited engagement with GVCs to the stringent private standards demanded by Northern lead firms (Interviews with seven producers, 2021). P3 had therefore recently opted to stop exporting via GVCs entirely, claiming that UK and EU retailers ‘only took the best fruit and left me stuck with the rest [of my harvest]’ (Interview, producer, P3, 2021). The producer group, P6-9, exported via P1. This producer group sold only a small percentage of its apples sporadically to Northern lead firms, a situation which helped to explain its inconsistent standards certification history.^10^ Given the small percentage exported to Northern lead firms and the costs associated with private standard certification, some members of the producer group were torn on whether certification against private social standards was worthwhile (Interviews, producers P8 and P9, 2021). Instead, both the producer group (P6-9) and P3 have found a still fairly lucrative, yet far less demanding buyer in the form of the discount arms of major South African retailers. Consequently, producers who very rarely supply Northern-oriented GVCs, or only supplied RVC and/or DVCs (particularly the discount arms of domestic retailers), are far less motivated to meet any private social standards. Compliance with labour regulation on supplier farms that only supply RVCs and DVCs is solely monitored by the severely under-resourced DoEL. While the rise of RVCs and DVCs offers smaller apple producers increased trading opportunities, it simultaneously exacerbates the regulatory vacuum associated with inadequately enforced labour regulation.

Farm-level findings indicated that the vast majority of permanent and temporary workers across all farming units—regardless of their market focus—reported compliance with certain aspects of labour regulation. This included having a signed contract (that was explained to them in their mother tongue); normal working hours being compliant with legislation; payment for overtime hours; and the presence and clear explanation of the farm’s sexual harassment policy.

More than 90% of workers – regardless of job status – also reported high levels of compliance with occupational health and safety standards. This included receiving health and safety training; access to trained first aiders, first aid kits, clean drinking water, clean toilets as well as handwashing facilities in the orchards. High overall levels of health and safety compliance across farms could be explained by PPECB’s proactive monitoring of process standards on all apple exporting farms (regardless of end-market destination) and acceptance of GlobalGAP, in lieu of the state’s own food safety regulation (GN707). That being said, higher rates of non-compliance with health and safety were reported by workers of producers who were GlobalGAP but *not* SIZA certified (P5 and P10) and, in the case of P10, not integrated into GVCs. This suggests that that the iterative, cumulative effect of complementary private standards (GlobalGAP and SIZA) implemented consistently and for a significant period of time, improved compliance with occupational health and safety standards on farms integrated into GVCs.

Serious labour legislation shortcomings were mostly found on producer sites principally oriented towards RVCs and DVCs, and felt most sharply by temporary workers on fixed-term contracts. Major areas of non-compliance with measurable standards were a lack of paid annual leave and paid sick leave for temporary workers. At two producer sites exporting only a small percentage of fruit to Northern lead firms, and thus adopting an inconsistent record of ethical certification (P7 and P9), all temporary workers interviewed reported not receiving paid annual leave (Interviews, 10 temporary workers, 2021). Due to a grey area in labour legislation—specifically SD13: 29(e) which stipulates that producers need only pay fixed-term workers leave if employed for more than four months—workers on short, fixed-term contracts are not *legally entitled* to paid leave. In principal, however, this practice remains arguably unethical and reflects the inadequate protection afforded by both private *and* public standards to temporary workers.

Regarding sick leave, temporary workers based at two production sites (P6 and 7), both SIZA certified, reported non-payment of sick leave (Interviews, 9 temporary workers, 2021). This finding was especially problematic, given workers were interviewed during the height of the Covid-19 pandemic. South Africa’s Covid regulations required that infected workers (or their contacts) must remain home-bound for a minimum of fourteen days. Non-payment of sick leave meant that workers either took unpaid sick leave and received no income for fourteen days, or, that ill and infected workers continued working to retain income. Three temporary workers at P6 reported that one Covid-positive manager returned to work and continued interfacing with workers, despite still displaying strong symptoms. Commented one of them:

‘*We are always scared we will get infected as there is no social distancing, the packhouse is always full and the manager comes to work while having tested positive and still looks sick*’ (Interview, temporary worker, P6, 2021).

Permanent workers’ complaints were about slightly higher-order issues, such as the state of on-farm housing; deductions related to such housing (Interviews, two permanent workers, P5 and P10, 2021); and their frustration about not being treated fairly:

Before the previous farm manager retired, I was told that his duties would be split between me and colleague and that his salary would be split between us as well. Now the manager is retired and his duties have been divided between the two of us but we have not received a pay rise (Interview, permanent worker, P10, 2021).

Major contraventions relating to less measurable standards such as discrimination, worker abuse and restrictions on freedom of association, were reported by temporary workers on sites only marginally integrated into GVCs and subject to inconsistent ethical certification (P6, 7, 8 and 9), and on sites not integrated into GVCs and therefore not ethically certified (P3). Issues reported included physical abuse:

*‘The manager shouts at us always and sometimes he hits us with a rod’ (Interview, temporary worker, P6, 2021);*

harsh behaviour:

*We are always terrified of the manager because he shouts at us all the time. There is a complaints box but we can’t even use it because we are scared we will not be hired in the next season should we voice our concerns. So because of a lack of employment in South Africa and us being uneducated, we just keep quiet and cry inside* (Interview, temporary worker, P6, 2021);

*We are forced to work in the rain, without access to rain clothes *(Interview, temporary worker, P7, 2021); and union dissuasion (Interview, two temporary workers, P3, 2021).

The overall lack of unionisation in the apple and wider horticulture sector was reflected in our findings, with P1 the only unionised farming operation. This was attributed to the fact that P1 had a DCA facility allowing it to run operations throughout the year and employ a larger component of permanent workers, who had more job security relative to those on fixed-term contracts; *and* the fact that P1 directly and consistently supplied large volumes of product to Northern lead firms via GVCs, rendering it subject to regular ethical audits and scrutiny, relative to other producer sites in our study (Interviews, two trade union representative, 2021). Contrastingly, the employment of mostly temporary workers on apple farms thwarts unionisation, as short, fixed-term contracts by their very nature are inherently job-insecure. As one temporary worker commented:

Management does not treat workers well. We are always scared and have to tread carefully as you might not be called to come to work again the next season… (Interview, temporary worker, P6, 2021).

In sum, the bulk of labour rights violations were reported by temporary workers, appointed on insecure, fixed-term contracts, subject to inadequate public and private regulatory protection and lacking trade union representation. Due to permanent workers being better protected by public and private regulation, and more job secure due to their contractual arrangements, experience and skill level, they reported less serious rights violations.

## Discussion and Conclusion

The governance-power framework deployed in this paper helps to explain key drivers of private–public governance across a complex myriad of intersecting GVCs, RVCs and DVCs. In Sect. “Private–public governance across intersecting apple value chains in South Africa” we examined private and public governance dynamics within each channel, before turning in Sect. “Public–private governance across intersecting value chains: implications for workers” to analyse their interactions across intersecting VCs and implications for workers. Drawing on the empirical findings above (summarised in Table 5), we are able to make a number of analytical contributions to existing GVC and related literatures.

**Table 5:** Private–public governance and power dynamics across value chains

	Private governance	Public governance	Public–private governance	Power
GVCs	Modular, driven by UK and EU lead firms through private standards including GlobalGAP and SIZA	All exporting producers must abide by ZA labour regulation and public export product quality and agricultural food safety regulation	SIZA reinforces social standards of Northern retailers and domestic labour and occupational health and safety (OHS) regulation. PPECB accepts GlobalGAP in lieu of its own food safety standards	D*yadic* and *direct* power linkages with standards shaped and enforced by lead firms
RVCs	Mixed governance: (1) Modular, driven by South African retailers, private governance only on product and food safety. But piggy-backing off global social standards (2) Arm’s length exchanges on wet markets; no private standards (visual inspection only). Inadvertent spill-over of social standards from certain sites serving GVCs	All exporting producers must abide by ZA labour regulation and public export product quality and food safety regulation	PPECB accepts GlobalGAP in lieu of own food safety standards Private and/or public labour standards are weakly enforced	Mixed power dynamics: (1) *dyadic* power in modular networks (while exerting *demonstrative* power to leverage GVC standards) (2) Lack of *dyadic* and/or *direct* power observed in arm’s length interactions with consolidators. Private governance underpinned by *collective institutional* (direct) and *constitutive* (diffuse) power, through the simultaneous enforcement of public regulations, and acceptance of private standards (e.g. GlobalGAP)
DVCs	Mixed governance: (1) Modular, driven by domestic supermarkets, private governance only on product and food safety. But piggy-backing off global social standards (2) Arm’s length exchanges on wet markets; no private standards (visual inspection only) Inadvertent spill-over of private social standards from certain sites serving GVCs	All producers must abide by ZA labour regulation and domestic product quality and food safety standards	Private and/or public labour standards are weakly enforced	Mixed power dynamics: (1) *dyadic* power in modular networks (while exerting *demonstrative* power to leverage GVC standards) (2) Lack of *dyadic* and/or *direct* power observed in arm’s length interactions with consolidators. Weak *collective, institutional* power of the state to enforce labour regulation

*Source* Authors’ own representation

Our findings indicate that in a context of polycentric trade and rising significance of RVCs and DVCs, analysis of governance and power must be understood in relation to the particular dynamics of VC intersection, specific to the particular value chain, institutional and country context in which production takes place. If GVCs are considered as a distinct and separate channel from other value chains (as per dominant conceptualisations, e.g. Gereffi, 1994; Gereffi et al., 2005; Ponte & Sturgeon, 2014), Northern lead firms’ direct and dyadic power can arguably be isolated in relation to their dedicated supplier base. Yet, expansion of RVCs and DVCs gives multi-chain suppliers a broader array of options, making them less beholden to their buyers, and potentially diminishing Northern lead firms’ dyadic and direct power to control production and exert standards over their suppliers. While existing analysis of governance and power has helpfully elucidated the diffusion of power *along* singular, Northern-oriented GVCs (see Dallas et al., 2019; Grabs & Ponte, 2019), our study highlights power diffusion *across* intersecting value chains—with significant and uneven implications for the public–private governance of labour standards.

On the one hand, our case shows that the growth of polycentric trade diffuses standards inherent to coordinated GVCs into RVCs and DVCs, conventionally understood as comprising arm’s length transactions absent of direct coordination. Beyond simply identifying spill-overs from GVCs into RVCs and DVCs (Barrientos et al., 2016; Krishnan, 2018; Pickles et al., 2016), we highlight three distinct and concurrent mechanisms through which diffusion of standards occur, involving private and public actors. First, this happens when social standards exerted by GVC-lead firms on their South African suppliers unintentionally spill over into RVCs and DVCs, served by the same GVC-oriented suppliers. Second, through the sourcing practices of DVC-lead firms who intentionally ‘piggy-back’ on Northern lead firms’ standards by purposefully sourcing from South African suppliers integrated into GVCs. This reflects a form of *demonstrative* power, as DVC-lead firms start ‘making strategic decisions based upon observation or mimicry’ of GVC-lead firms’ behaviour (Dallas et al., 2019:16), resulting in social standards being informally transmitted not along, but *across* GVCs to RVCs and DVCs. Third, by directly exerting collective, institutional power to *intentionally* enforce food safety regulation, PPECB *unintentionally* diffuses private social standards when accepting GlobalGAP in lieu of its own food safety regulation, thus reflecting a more diffuse form of *constitutive* power. As previously discussed, GlobalGAP is a taken-for-granted, best practice private standard that also incorporates occupational health and safety standards, thereby benefitting farm workers.

On the other hand, the growth of polycentric trade has generated a concurrent ‘private–public governance void’ of labour standards. This is largely due to the heterogeneous purchasing practices of Southern lead firms sourcing via RVCs/DVCs. While they sometimes (intentionally) piggy-back on private social standards by sourcing from GVC-integrated (and certified) suppliers, they also commonly source from non-GVC-integrated producers and NFPMs (where no private standards apply), due to seasonal and price preferences, such as for their discount stores where reputational risk is perceived as less critical relative to competitive pricing. We observed a ‘private governance void’ on supplier sites minimally oriented to GVCs, and heavily integrated into RVCs/DVCs where social standards were not applied. This was compounded by inadequate levels of public governance protection, due to the state’s inability to enforce labour legislation across all fruit production sites under study.

All of this has significant implications for the regulatory protection and employment conditions of *workers* operating across intersecting value chains. Stemming from the above observations, our study found non-compliance with labour rights was more serious on farms less-oriented to supplying GVCs and therefore less subject to private social standards, relative to farms that were GVC-oriented, ethically certified and subject to regular audits. In line with previous studies, the bulk of labour rights violations were reported by temporary workers, whose contracts are far more precarious relative to permanent workers. Temporary workers are appointed on fixed-term contracts associated with high job insecurity, insufficient public and private regulatory protection and a lack of access to trade union representation. Permanent workers mostly reported less serious violations, possibly due to a combination of labour legislation (which provides them with better protection relative to fixed-term workers), and their relative job security, stemming from their permanent contracts, knowledge, training and skill levels which are critical in ensuring compliance with a raft of private standards across multiple end-markets. Given the increasing importance of RVCs and DVCs relative to GVCs, this is a key contribution to existing literature on labour standards and business ethics that, to date, has focused primarily on North–South GVCs.

In making these points we fully acknowledge that private social standards in GVCs contain significant weaknesses. They are drafted by powerful private actors in far-flung locations; often fail to address more critical and less easily measured working conditions at point of audit (i.e. protection from discrimination; right to freedom of association); provide insufficient protection to temporary workers and are stymied by lead firms’ contradictory demands for high-quality, low-cost goods with short lead times (Alford, 2020; Barrientos & Smith, 2007; Lund-Thomsen & Lindgreen, 2014). However, they do offer a crucial layer of regulatory protection for two key reasons. First, Northern lead firms are held accountable by civil society organisations and consumers, to leverage their dyadic and direct power over producers to ensure compliance with labour standards (Barrientos, 2019). Second, compliance with private standards (such as SIZA) often requires adherence to national, public labour regulations in producing countries (Alford, 2016), meaning private codes can ‘complement’ enforcement of public regulations (Bartley, 2018; Locke, 2013). We would therefore encourage further exploration into the nature and form of private and public labour standards across intersecting value chains, and their implications for workers in different sectors and country contexts.

## Data Availability

The data supporting the findings of this study is available on request to the authors and in line with the guidelines of the Journal of Business Ethics. The study data is however not publicly available due to restrictions that were conditional to obtaining ethical clearance from the authors’ affiliated institutions.

## Footnotes

1 To clarify, ‘global value chains’ refer to traditional sourcing arrangements driven by transnational retailers located in the global North, sourcing from suppliers in the global South. ‘Regional value chains’, concern buyer–supplier relationships that are contained within one world region, such as Africa. ‘Domestic value chains’ refer to sourcing wherein the relationship between the buyer and supplier is confined to one country.

2 SIZA is informed by the ETI base code, ILO conventions as well as South African legislation, thus reinforcing South African workplace regulation which is poorly enforced by the state.

3 Of the rest of the 2019-crop, 45% was exported, 30% went for juicing, while a negligible percentage was sold as dried fruit (Hortgro: ‘Key Deciduous Fruit Statistics’, p16, available online at: https://www.hortgro.co.za/markets/key-deciduous-fruit-statistics/).

4 Producer-exporters interviewed sold 5–50% of their crop on NFPMs.

5 The dominant South African supermarkets, in order of the number of stores, are Shoprite, Pick n Pay, Massmart and Spar. The local supermarket sector is so significant that in 2020 four of South Africa’s major supermarkets made it into Deloitte’s Global Powers of Retailing list, which identifies the 250 largest retailers around the world. Available online at: https://www2.deloitte.com/content/dam/Deloitte/at/Documents/consumer-business/at-global-powers-retailing-2020.pdf).

6 Only one upmarket supermarket consistently demand compliance with social standards (Interviews, four exporters, 2019–20). Two other supermarket groups require private social standards when sourcing product on its *branded, in-house products* only.

7 Email correspondence with SIZA representative, 2020.

8 For example, resource constraints meant that just 23% of inspections conducted by IES in the Western Cape targeted the agriculture, forestry and fishing sector.

9 PPECB is an assignee of South Africa’s Department of Agriculture, Land Reform and Rural Development (DALRRD).

10 P6 was ethically certified from 2000 to 2010, but from 2011 to 2015 it stopped exporting to Northern lead firms and its ethical certification lapsed. It was only in 2016 that P6 became SIZA recertified. P9 was certified against the SIZA-standard in 2015, then left the packhouse group and was only recertified again when it re-joined the group in 2019; P8 was certified in 2018 and P7 was certified in 2020.
